# Expression Analysis of *Moritella viscosa*-Challenged Atlantic Salmon Identifies Disease-Responding Genes, MicroRNAs and Their Predicted Target Genes and Pathways

**DOI:** 10.3390/ijms231911200

**Published:** 2022-09-23

**Authors:** Sigmund Ramberg, Aleksei Krasnov, Duncan Colquhoun, Christian Wallace, Rune Andreassen

**Affiliations:** 1 Department of Life Sciences and Health, Faculty of Health Sciences, OsloMet-Oslo Metropolitan University, 0167 Oslo, Norway; 2 Division of Aquaculture, Norwegian Institute of Fisheries and Aquaculture (Nofima), 1430 Ås, Norway; 3 Norwegian Veterinary Institute Ås, 1433 Ås, Norway; 4 Veso Vikan, 7810 Namsos, Norway

**Keywords:** winter-ulcer, disease response, microRNA, small-RNA sequencing, Atlantic salmon, head kidney, microarray, transcriptome

## Abstract

*Moritella viscosa* is a bacterial pathogen causing winter-ulcer disease in Atlantic salmon. The lesions on affected fish lead to increased mortality, decreased fish welfare, and inferior meat quality in farmed salmon. MicroRNAs (miRNAs) are small non-coding RNAs involved in post-transcriptional regulation by guiding the miRNA-induced silencing complex to specific mRNA transcripts (target genes). The goal of this study was to identify miRNAs responding to *Moritella viscosa* in salmon by investigating miRNA expression in the head-kidney and the muscle/skin from lesion sites caused by the pathogen. Protein coding gene expression was investigated by microarray analysis in the same materials. Seventeen differentially expressed guide-miRNAs (gDE-miRNAs) were identified in the head-kidney, and thirty-nine in lesion sites, while the microarray analysis reproduced the differential expression signature of several thousand genes known as infection-responsive. In silico target prediction and enrichment analysis suggested that the gDE-miRNAs were predicted to target genes involved in immune responses, hemostasis, angiogenesis, stress responses, metabolism, cell growth, and apoptosis. The majority of the conserved gDE-miRNAs (e.g., miR-125, miR-132, miR-146, miR-152, miR-155, miR-223 and miR-2188) are known as infection-responsive in other vertebrates. Collectively, the findings indicate that gDE-miRNAs are important post-transcriptional gene regulators of the host response to bacterial infection.

## 1. Introduction

The Atlantic salmon (*Salmo salar*) is a species of great interest to the aquaculture industry [1], but despite advancements in vaccination and non-medicinal pest management techniques, infectious diseases remain a major cause of reduced production quality and loss for farmed salmon, as well as an ongoing welfare problem for the fish [2]. Increased infection pressure on wild populations from farmed salmon is also a persistent public concern, although pathogen prevalence in wild populations remains difficult to study [3,4]. One of these pests is the bacterial pathogen *Moritella viscosa*, which causes winter-ulcer disease, leading to open lesions on the sides of affected fish, typically during the winter months [5,6,7]. These ulcers lead both to increased mortality, and to rejection or reduced quality grade of fish at the time of slaughter [8]. While different commercial vaccines against the disease have been available for several decades, none of these provide complete protection against infection [9,10], and the disease remains both a significant source of economic loss in aquaculture, and reduced quality of life for farmed salmon [8].

MicroRNAs (miRNAs) are short (usually ~22nts) single-stranded non-coding RNAs which are key molecules that select which protein coding transcripts should be controlled by negative post-transcriptional gene regulation mechanisms. Such regulation has been shown to affect a diverse set of biological processes [11,12]. miRNA activated post-transcriptional regulation is also involved in immune responses to both viral and bacterial diseases in vertebrates [13,14]. Initially, microRNAs are transcribed as long primary miRNAs (pri-miRNAs), which undergo multiple processing steps to reach their functional form [15]. Hairpin structures containing precursor miRNAs (pre-miRNA) are generated from the pri-miRNA by the Drosha-containing microprocessor complex cropping off extraneous sequence. These pre-miRNAs are exported to the cytoplasm, where the loop structure is removed by Dicer, leaving a short miRNA duplex [16]. One of the strands of the duplex, designated the guide miRNA, becomes part of the miRNA-induced silencing complex (miRISC) along with Dicer, TRBP, PACT and Argonaute proteins. The other strand (called passenger miRNA) is typically degraded, often leading to much lower abundance of this mature miRNA than the biologically relevant counterpart produced from the same precursor [17,18,19,20]. The guide miRNA initiates repression of translation or degradation of a transcript (termed the target transcript) by guiding the miRISC complex to bind to it, most commonly in the 3′-untranslated region of protein coding transcripts [21].

Following the initial characterization of miRNA genes in Atlantic salmon [17], there has been a recent expansion of the known miRNAome [18]. A publicly available high quality full-length Atlantic salmon mRNA transcriptome [22] (Genbank Accession GIYK00000000) has also facilitated the generation of a comprehensive resource of predicted miRNA target transcripts (https://github.com/AndreassenLab/MicroSalmon/ (accessed on 20 September 2022)) [23]. This has led to Atlantic salmon being the aquaculture species with the most comprehensive resources available for differential expression studies of miRNAs and their putative target genes. Differential expression of particular miRNAs in response to infection by viral, bacterial and parasitic pathogens indicates that some miRNAs are involved in disease response [24,25,26,27,28,29]. Significant losses of life are also experienced during smoltification, the process during which Atlantic salmon adapt to life in seawater. Similar expression studies examining the response to smoltification indicate that post-transcriptional regulation by miRNAs is also involved in facilitating that developmental transition [30,31]. Associations between differentially expressed miRNAs (DE-miRNAs) and infectious disease have been reported for several economically important pathogens in Atlantic salmon. Some of these include Salmonid Alphavirus [25], Infectious Pancreatic Necrosis Virus [26], Infectious Salmon Anemia Virus [27], *Piscirickettsia salmonis* [28], as well as sea louse infestation [29].

Characterization of miRNAs responding to disease may provide a better understanding of the mature miRNAs involved in immune or stress responses, as well as indicate which protein coding genes and genes pathways they regulate. Such miRNAs are also potential biomarkers for marker-assisted breeding, which may aid in production of healthier and more resistant salmon strains for farming. Better understanding of the regulatory activities of salmon miRNAs could also aid in development of functional feeds [32] or be applied as therapeutics for disease control [33,34]. So far, the role of miRNAs in response to *M. viscosa* infection in Atlantic salmon has not been characterized. Therefore, the aim of this study was to characterize miRNAs and mRNAs that show differential expression behavior in response to *M. viscosa* challenge in skin and dermal muscle tissue sampled from fish infected by *M. viscosa* and graded by severity of lesions. In parallel, expression analysis was also carried out in head-kidney, which is an important immune organ in teleost fish. Unlike miRNAs, mRNAs responding to *M. viscosa* infection have been previously characterized in Atlantic salmon by microarray analysis or high-throughput sequencing [6,35,36,37]. The results of these studies (differentially expressed mRNAs) have been used as input in enrichment analyses to reveal biological processes that are affected by infection. Such enrichment analyses rely on functional annotation of the differentially expressed mRNAs (DE-mRNAs), which in turn are mostly based on their homology with well-annotated genes from higher vertebrates. Another approach for annotation of differentially expressed genes, introduced by Krasnov et al. [36], does not annotate DE-mRNAs by assumed function inferred from their homology to evolutionary distant species. Instead, genes are grouped into Transcription Modules (TMs) by showing consistent co-regulation in response to different factors (viral infection, bacterial infection, inflammation, or stress) across a large set of microarray studies. This approach assigned genes as responding to certain pathogens or biological processes based on their expression behavior in similar, independent expression studies rather than their inferred functions based on their nucleotide sequences. This approach avoids assumptions about preserved functionality of homologous genes across phylogenetically distant species. It also allows for classification of genes unique to teleosts or genes with unknown functions to be assigned to TMs. This, in turn, allows for the detection of genes that respond to, e.g., viral infection or bacterial infection by carrying out enrichment analysis of DE-mRNAs against the TMs classified in prior meta studies. This approach has the advantage that it detects genes important to the conditions studied that may not have been revealed by the common homology-based enrichment analysis. Here, we explore the *M. viscosa*-responding genes identified by microarray analysis by common homology-based enrichment analysis as well as TM based enrichment analysis. The subset of the DE-mRNAs which were predicted to be targets of the disease-responding miRNAs (tDE-mRNAs) were also analyzed using both the expression behavior derived TM framework and annotation by homology-based frameworks. The enrichment analysis of this subset of DE-mRNAs (tDE-mRNAs) could reveal gene pathways that are likely to be controlled by the miRNAs responding to *M. viscosa* infection.

## 2. Results

### 2.1. M. viscosa Challenge, Lesion Scores, RNA Extraction and Moritella qPCR Measurements

A total of 46 samples, 22 from the head-kidney (HK), and 24 from lesion sites (LS), were collected at various time points (0, 9, 23 and 34 days post-challenge (DPC)) throughout the *M. viscosa* challenge trial. An overview of concentration, quality measurements and measurements of infection by qPCR in the 22 individual HK samples is given in Table 1. The concentrations, absorbance ratios and RNA Integrity Number (RIN) value measurements showed that all RNA extracts included were of satisfactory quality for qPCR and small RNA sequencing. The number of raw reads from these samples following small-RNA sequencing ranged from 3.8 to 15.4 million. Following adapter trimming and size filtering, between 1.2 million and 6.9 million reads were mapped onto the reference miRNAome. All small RNA sequenced samples have been submitted to the Sequence Read Archive, NCBI, and the accession numbers are given in Table 1. All HK samples were analyzed by *M. viscosa* qPCR to measure degree of infection by the bacteria in HK (Table 1). These results showed that *M. viscosa* was not detected in HK nine DPC (Time Point 1, T1), despite being identified in LS samples from the same fish. Lesion scores were also recorded for the individual fish from which each sample originated. A few of the fish from T1 showed dermal lesions caused by *M. viscosa* infection. Conversely, the qPCR showed that all fish were infected with *M. viscosa* in HK from Time Point 2 (T2, 23 DPC) onwards. However, the CT-values from both T2 and Time Point 3 (T3, 34 DPC) indicated a moderate infection in HK, which did not seem to increase over time or show strong correlation with lesion scores. 

A summary of the concentration, quality measurements and qPCR results from the 24 individual LS samples is shown in Table 2. Again, the measurements indicated high quality of all samples that were subsequently used for small-RNA sequencing. The number of raw reads from these samples following small-RNA sequencing ranged from 7.5 to 13.3 million. Following adapter trimming and size filtering, between 1.3 million and 7.0 million reads were mapped onto the reference miRNAome. Sequence Read Archive accession numbers for all samples are given in Table 2. Unlike the HK samples, all challenged LS samples were positive. However, the measured CT-values seemed to vary greatly within each time-point, and samples with the most severe lesion score (2) were associated with a low CT-score while time point groups did not show such an association. Because of this, these samples were instead grouped by range of measured CT-score rather than time points in the differential expression analysis (Section 2.2). All CT < 20 samples displayed lesions that penetrated the basement membrane (lesion score 2). Two of the samples from the mid-level group (CT 20–30) showed mild lesions (lesion score 1). One of the fish from the high CT group (CT > 30) showed mild lesions, while the others had no visible lesions. 

Some additional samples collected both among HK and LS were initially RNA extracted but did not meet our quality criteria. These were therefore not included in downstream analysis, and the sample sets shown in Table 1 and Table 2 were those used in the analysis of differential expression (Data on samples not used is not shown). Some LS control samples were among those not included, reducing controls to three samples in this tissue. The control samples collected at 9 DPC were, however, from the same time point as fish revealing medium to severe infection when analyzed by qPCR. 

### 2.2. DESeq Analysis of miRNA Expression

#### 2.2.1. Changes in miRNA Expression in Lesion Sites during Infection

**Figure 1:**
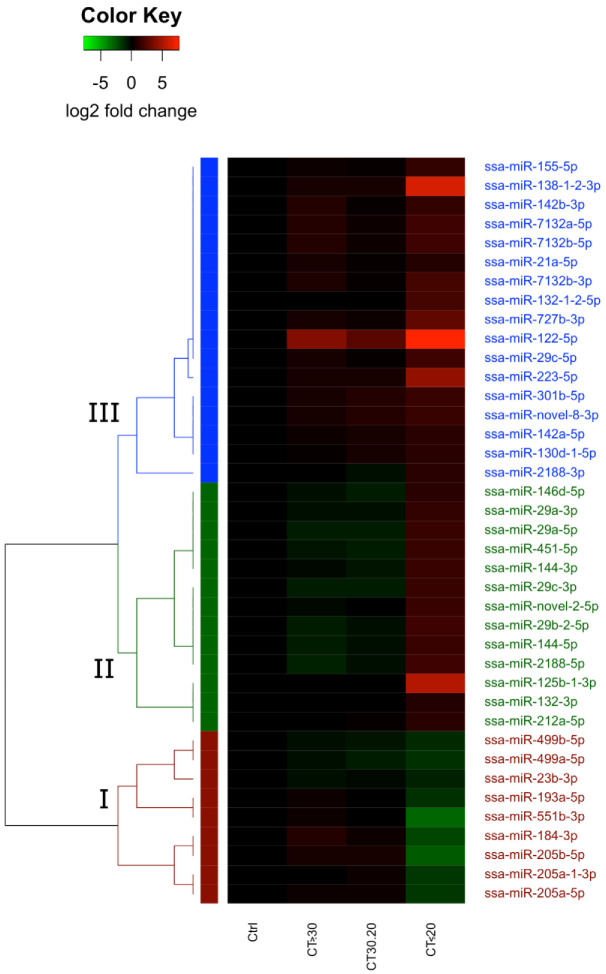
Heatmap showing results from clustering analysis of the gDE-miRNAs in the LS materials. Each line represents one gDE-miRNA, with the colour in each row representing the log2 fold change in abundance when compared to the control group. Major clusters of gDE-miRNAs are annotated as I (Cluster 1, red), II (Cluster 2, green), and III (Cluster 3, blue).

The control group (LS Control) was compared to each of the low, medium, and high infection level groups (CT > 30, CT 20–30, and CT < 20), described in Table 2, to identify miRNAs that differ in their expression depending on the infection severity. A total of 63 unique mature DE-miRNAs were identified across the three sample groups. A complete overview of all DE-miRNAs is included in Supplementary Table S1. Of these, 39 were denoted as guide DE-miRNAs (gDE-miRNAs), meaning that if one of the two mature miRNAs originating from the same precursor showed >10× abundance, only that one was considered to be a guide miRNA (see methods). One gDE-miRNA (ssa-miR-122-5p) was discovered both in the comparison with the low infection group (CT > 30) and the high infection group (CT < 20), while all others displaying significant differential expression were identified in the CT < 20 group. Thus, the results showed strong correlation between severe infection/lesion scores and differential expression of miRNAs. Hierarchical clustering analysis with the expression changes of each of the 39 gDE-miRNAs as input was carried out to identify gDE-miRNAs with similar patterns of expression change across the LS groups compared. The analysis revealed three major clusters of gDE-miRNAs. The results are illustrated in the heatmap shown in Figure 1 and the gDE-miRNAs in each cluster are shown in Table 3.

Cluster 1 (I, red letters in Figure 1) consisted of nine gDE-miRNAs from six families. These followed a pattern of very low change in expression in the low and medium infection groups (five weakly downregulated and four weakly upregulated), and strong downregulation in the high infection group. Cluster 2 (II, green letters in Figure 1) consisted of 13 gDE-miRNAs from nine families. No changes or weak downregulation were identified in the low and medium infection groups, but sharp upregulation was identified in the high infection group. Cluster 3 (III, blue letters in Figure 1) consisted of 17 gDE-miRNAs from 14 families. In contrast to Cluster 2, these showed weak, although non-significant, upregulation in the low to medium infection groups, indicating a higher sensitivity to infection. However, like Cluster 2, these miRNAs also showed a much larger increase in their expression in the highly infected group (CT < 20).

Clusters 2 and 3 each included one miRNA putatively unique to Atlantic salmon (ssa-miR-novel-2-5p and ssa-miR-novel-8-3p, respectively), providing insight that these salmon specific miRNAs are disease-responding miRNAs. All family members showed very similar expression changes, and all members within a family were grouped together as being either co-downregulated or co-upregulated at CT < 20.

#### 2.2.2. Changes in miRNA Expression in the Head-Kidney during Infection

The comparisons between the control group (HK Control) and the three time point groups (T1, T2, and T3) described in Table 1 reveal a total of 26 unique DE-miRNAs. A complete overview of all DE-miRNAs is included in Supplementary Table S1. Seventeen were annotated as gDE-miRNAs. The majority showed significant expression changes in groups T1 and T3. Six of the miRNAs (ssa-miR-1-3p, ssa-miR-15bf-5p, ssa-miR-23c-3p, ssa-miR-133-1-4-5-3p, ssa-miR-133-2-3-3p, and ssa-miR-206-3p) did, however, show significant differences in expression at multiple time points. An overview of all gDE-miRNAs is shown in Table 4.

**Figure 2:**
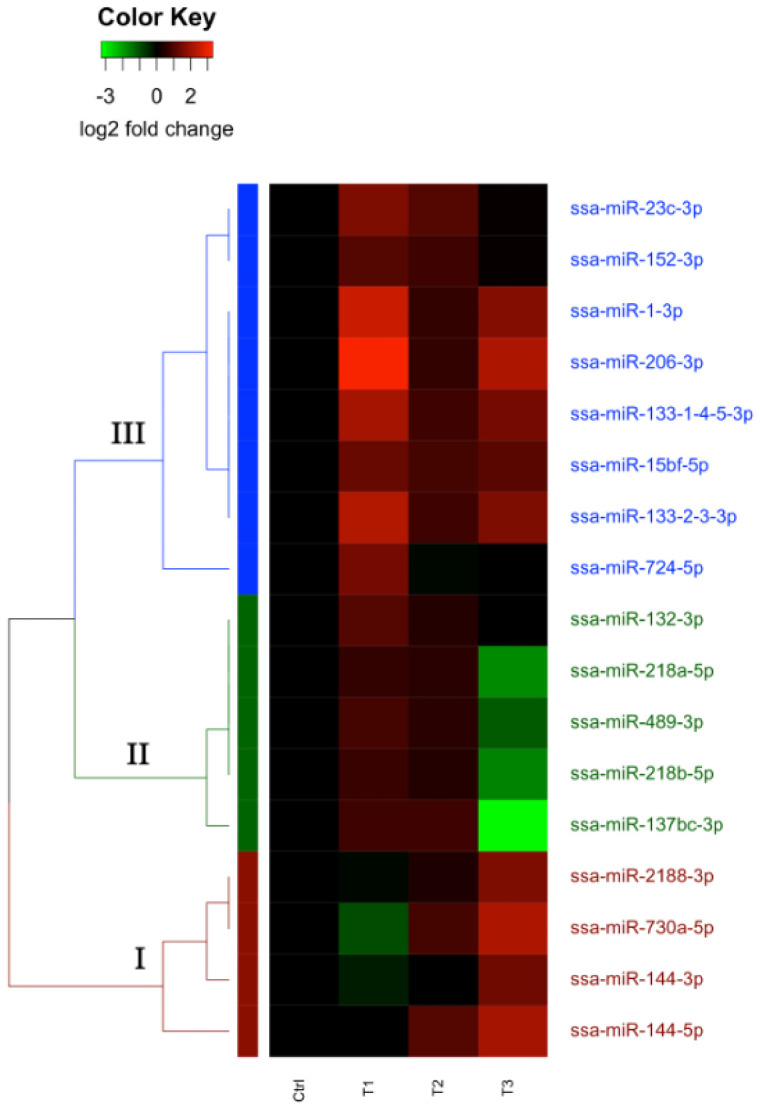
Heatmap showing results of clustering analysis for gDE-miRNAs based on changes in the HK samples grouped by sampling time point. Each line represents one miRNA, with the colour in each row representing the log2 fold change in abundance when compared to the control group. Major clusters of gDE-miRNAs are annotated as I (Cluster 1, red), II (Cluster 2, green), and III (Cluster 3, blue).

Hierarchical clustering analysis of the 17 gDE-miRNAs revealed three major clusters based on their patterns of expression change, illustrated in Figure 2. All of the gDE-miRNAs showed weak upregulation at T2, and the main differences between clusters were based on the changes at T1 and T3.

Cluster 1 (I, red letters in Figure 2) consisted of four gDE-miRNAs from three families. The miRNAs in this cluster were all weakly downregulated and upregulated at T1 and T2, respectively, but strongly upregulated at T3. These miRNAs thus displayed a late response to infection. Cluster 2 (II, green letters in Figure 2) consisted of five gDE-miRNAs from four families. The miRNAs in this cluster also showed a late response to infection, but in contrast to cluster 1, showed a substantial downregulation at T3. In contrast, Cluster 3 (III, blue letters in Figure 2) consisted of eight gDE-miRNAs from seven families, which were all strongly upregulated at T1 and slightly less but still strongly upregulated at T3. The gDE-miRNAs in this cluster, thus showed a relatively rapid response to infection despite the pathogen not being detected in HK at this time point.

Initially, 63 and 26 DE-miRNAs were identified in LS and HK, respectively, but only 39 and 17 of these were manually annotated as gDE-miRNAs. For the majority of the DE-miRNAs, only one mature miRNA of the two processed from same precursor was classified as a guide miRNA when applying our read count thresholds (>10 difference). Interestingly, while multiple gDE-miRNAs were identified in each of the HK and LS tissues that responded to *M. viscosa* infection, there was very little overlap between which specific miRNAs were identified in both groups. This was the case even if the HK analysis was alternatively, like the LS analysis, carried out with only the three T1 controls. Only four miRNAs (ssa-mir-132-3p, ssa-mir-144-3p, ssa-mir-144-5p, and ssa-mir-2188-3p) were gDE-miRNAs in both tissue types. Furthermore, these were all upregulated in response to the infection in both tissues. Despite responding to the same infection, the remaining gDE-miRNAs were differentially expressed in only one of the tissues, indicating tissue specific regulatory roles.

### 2.3. Discovery of Differentially Expressed mRNA Transcripts Using Micro-Array Analysis

A 44k salmon-specific mRNA microarray (Salgeno-2) was used to identify differentially expressed mRNAs (DE-mRNAs) in HK and LS materials. A total of 1568 oligos in the head-kidney materials and 4217 oligos in the lesion site materials were detected as differentially expressed in the microarray analysis (Supplementary Table S2). The results from the differential expression analysis were further processed by the STARS pipeline [38], which performed enrichment analysis of the DE-mRNAs applying two different main approaches. One based on homology-based functional annotation of the genes detected by individual oligos, in the GO and KEGG frameworks, as well as the STARS framework, which is designed specifically for fish [38]. The other approach was based on the TM/TS framework, as described in the introduction, methods (Section 4.5) and Krasnov et al. [36]. Applying this approach, the DE genes are categorized based on their expression patterns in this study compared to patterns in other similar microarray studies. This approach identified genes belonging to a Transcription Module (TM), which is a set of genes that are co-regulated in response to the same condition across multiple studies, or to a Transcription Signature (TS) which is a set of genes that were co-regulated in a specific study. The complete results from the STARS pipeline’s analysis, as well as the microarray readings for each individual gene, are shown in Supplementary Table S2. The main findings of the TM/TS and STARS enrichment analyses are given in Table 5, Table 6 and Table 7. 

Table 5 gives an overview of highly enriched TM categories along with a selection of TS categories of interest for both LS and HK samples. For the TMs, both groups showed significant (>7 times expected value) enrichment for genes upregulated in response to bacterial infection, while the LS samples additionally showed enrichment (>6 times expected value) for genes associated with inflammation across previous microarrays.

The TS categories followed the trend suggested by the TMs, and the similarities with prior studies were highest in genes upregulated in response to bacterial infection in both materials, including *Moritella viscosa*, as well as inflammation-causing muscular stressors. In LS, these also included the categories plasmid injection, wound healing, and pro-inflammatory response in skeletal muscle to an astaxanthin-free diet. The LS genes also showed significant enrichment of genes responding to *Salmonid alphavirus*, suggesting many of these were immune-response genes activated in both viral and bacterial infections. 

In addition to enrichment in TS categories responding to bacterial infections, vaccination, and simulated infection using poly (I:C) in HK, there was also significant enrichment of genes responding to acute stress from exhaustion swimming, or adaptation to seawater. Lastly, there was significant enrichment of genes in the TS category responding to induced erythropoiesis, suggesting the head-kidney plays a role in hemostasis, associated with bleeding caused by winter-ulcer lesions, supporting earlier findings in teleost fish [46,47].

Table 6 and Table 7 show selected enriched functional categories from analysis in the STARS framework involved in immune processes or metabolism and tissue growth, along with mean log2-fold change for the genes in each category in the LS and HK materials, respectively. In LS (Table 6), there was a significant upregulation of a wide array of immune-responsive genes, including cytokines, antigen presentation, tumor necrosis factor-related genes, and lymphocyte activation, among others. There was also upregulation of genes related to blood coagulation, cellular stress, and protein folding. One surprising finding was that genes in the functional category xenobiotic metabolism, which includes the breakdown of toxins, was downregulated in these materials. Additionally, there was a broad downregulation of genes related to cellular growth and metabolism, with the exception of lysosomes and proteasomes, indicating that many of the normal cell functions in the infected and wounded muscle tissues are suppressed while they are fighting the infection and dead cells are being degraded [48]. 

Many categories of immune-relevant genes were downregulated in the HK materials at one or multiple time points (Table 7). Many of these were the same categories that revealed mean upregulation in the LS materials. Two notable exceptions to this trend were protein folding and modification, which was upregulated in both materials, and xenobiotic metabolism, which is upregulated in HK but downregulated in LS. The difference in the latter category suggests that the head-kidney plays a role in response to bacterial toxins. Furthermore, both Glutathione metabolism and redox homeostasis are upregulated at Time Point 3, suggesting a role in response to oxidative damage to tissues. 

Lastly, at Time Point 3, and sometimes at earlier time points, there was an upregulation of genes related to cell growth, particularly keratin and myofiber, as well as genes involved in erythropoiesis, supporting the TS findings and prior studies indicating that the head-kidney plays a role in hemostasis [46,47]. Of note, genes related to Globins were strongly upregulated at Time Point 3, but were significantly downregulated at earlier time points, indicating a sudden shift in the regulation of these hemostasis-related pathways.

These results agree well with previous microarray studies on *Moritella* [36]. This indicates that gene expression changes both in DE-mRNAs as well as gDE-miRNAs are representative for responses to *M. viscosa* infection. Consequently, the following characterization of gDE-miRNAs in terms of their predicted targets and resulting functional annotation should also be representative for their role in response to *M. viscosa* infection.

### 2.4. In Silico Prediction of gDE-miRNA Targets (tDE-mRNAs) and Overrepresentation Analysis

The function of gDE-miRNAs was further explored by predicting their targets among the DE-mRNAs (tDE-mRNAs). Enrichment analysis of these target genes was then applied to reveal overrepresented TM/TS categories while gene ontology-based enrichment analysis was applied to reveal functional pathways associated with the targets of the gDE-miRNAs. 

Initially, the sequences from oligos on the microarray which did detect a DE-mRNA were utilized to identify the corresponding full-length mRNAs (FL-mRNAs)(see methods), in the current Atlantic salmon FL-transcriptome [22]. This was necessary to retrieve the 3′UTRs needed for the target prediction analysis. The oligo sequences from the HK analysis identified on average 2.7 FL-mRNAs each while the oligo sequences from the LS analysis identified on average 2.9 FL-mRNAs each. These FL DE-mRNAs represented isoforms of the DE-mRNAs. 

The FL DE-mRNAs and the gDE-miRNAs were then used as input in the MicroSalmon database (https://github.com/AndreassenLab/MicroSalmon/ (accessed on 20 September 2022)) [23] to retrieve the predicted tDE-mRNAs. Roughly two thirds of the FL-mRNAs were identified as a target (tDE-mRNAs) of at least one gDE-miRNA based on the in silico predictions of target genes. The final number of genes following manual annotation was slightly lower than the number of loci, as several highly similar paralogous loci were annotated as encoding the same gene. Table 8 gives an overview of the results from the in silico prediction of target genes showing number of FL DE-mRNAs in the HK and LS materials corresponding to different isoforms of a DE-mRNA, number of tDE-mRNAs among the FL DE-mRNAs and the final set of different genes. 

A complete overview of predicted tDE-mRNAs along with the individual gDE-miRNAs targeting each transcript, gene names and symbols, transcript accession numbers and GO terms is given in Supplementary Table S3 for HK samples and Supplementary Table S4 for LS samples.

The roles of the gDE-miRNAs were further explored by enrichment analysis of their tDE-mRNAs. As in the enrichment analysis of the DE-mRNAs (Section 2.3), the two approaches TM/TS enrichment and gene ontology-based enrichment were applied for this purpose. 

The tDE-mRNA were fed back into the STARS pipeline to identify enriched TM/TS categories [36]. The advantage of this approach was that it did not depend on functional annotation of the tDE-mRNAs (109 tDE-mRNAs did not have any functional annotation). The results for TM and the highly enriched TS categories in LS materials and HK materials are shown in Table 9. The complete set of significantly enriched TM/TS categories are shown in Supplementary Table S5.

Comparing the enrichment scores in Table 5 (DE-mRNAs) and Table 9 (tDE-mRNAs), there was very similar results, with a slight increase in enrichment for most categories in the enrichment analysis of tDE-mRNAs. In summary, this indicated that the gDE-miRNAs were predicted to regulate genes that are known to be involved in response to bacterial infection (*M. viscosa* infection), inflammation, wound healing and erythropoiesis (Table 9). 

Following functional annotation of tDE-mRNAs by utilizing the information from their FL-mRNA coding sequences (see methods), they were used as input in the more commonly applied gene ontology-based enrichment analysis. The non-redundant lists of the 551 genes used as input from HK and the 1917 from LS, along with the complete results of the PANTHER overrepresentation analysis from both materials are shown in Supplementary Table S6 and listed by targeting gDE-miRNAs in Table S7. 

**Figure 3:**
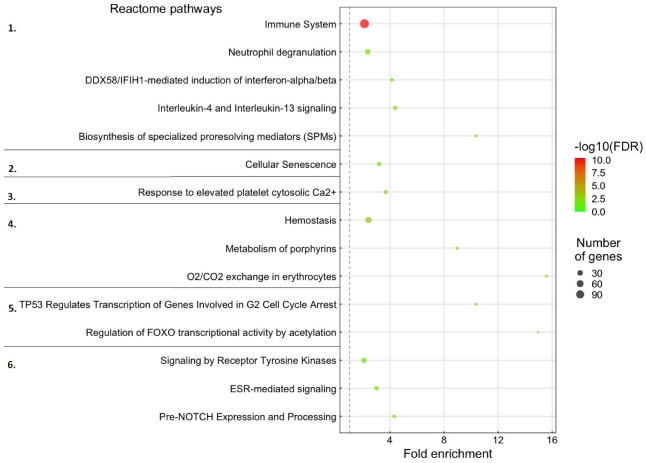
Enriched gene pathways associated with tDE-mRNA genes in head-kidney. The dot size indicates the number of tDE-mRNA genes associated with the process and the dot color indicates the significance of the enrichment (−log10 FDR-corrected *p*-values). The dot’s position on the Y-axis indicates fold-enrichment compared to the expected occurrence for a random set of genes of that size. The Reactome pathways were subdivided into the following categories indicated by the numbers on the far left: 1. Immune system. 2. Response to Stress. 3. Platelet Activation. 4. Hemostasis. 5. Cell Cycle Control. 6. Receptor Signaling.

The most specific enriched gene pathways in HK (Reactome pathways) are shown in Figure 3. There were multiple immune system pathways among the enriched categories including production of both pro-inflammatory, anti-inflammatory, and pro-resolving proteins. The most highly enriched immune pathway was Biosynthesis of specialized pro-resolving mediators (SPMs) at 11.68 times fold change. Other pathways with obvious relevance to winter-ulcer were those involved in production of blood and response to bleeding. Gene pathways involved in stress responses, cell cycle control and receptor signaling were also highly enriched for tDE-mRNAs with O_2_/CO exchange in erythrocytes (15.57 times fold change) as the one showing largest enrichment.

**Figure 4:**
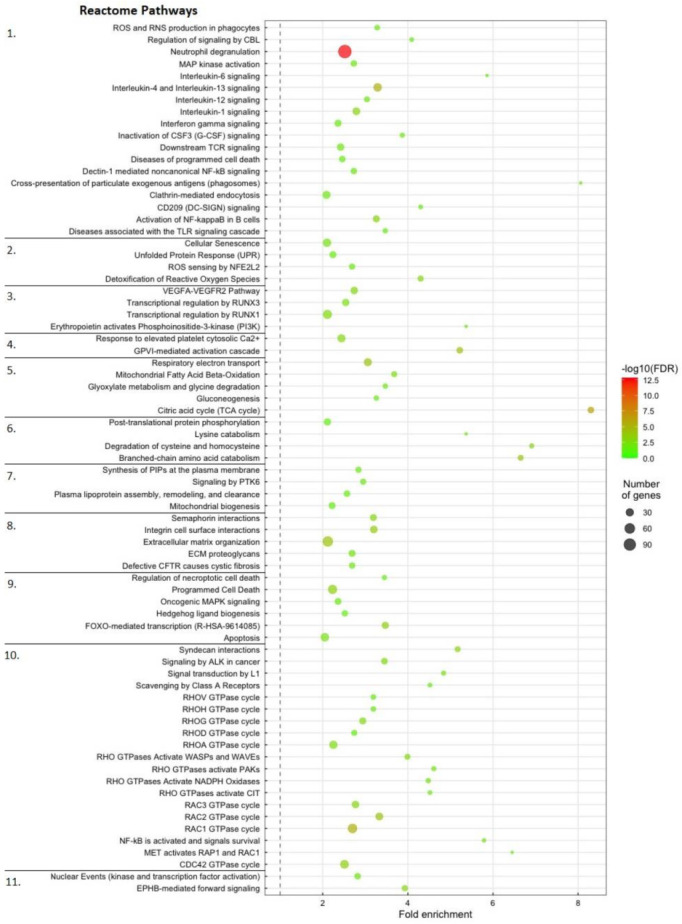
Enriched gene pathways associated with tDE-mRNA genes in lesion sites. The dot size indicates the number of tDE-mRNA genes associated with the process and the dot color indicates the significance of the enrichment (-log10 FDR-corrected *p*-values). The dot’s position on the Y-axis indicates fold-enrichment compared to the expected occurrence for a random set of genes of that size. The Reactome pathways were subdivided into the following categories indicated by the numbers on the far left: 1. Immune System. 2. Response to Stress. 3. Angiogenesis. 4. Platelet Activation. 5. Energy Metabolism. 6. Metabolism of Amino Acids. 7. Cell Maintenance. 8. Cell Communication. 9. Cell Cycle Control. 10. Receptor Signaling. 11. Neuronal Growth.

While the most specific enriched Reactome pathways in LS (Figure 4) also included pathways related to immune response, stress and bleeding observed in HK, the additional pathways in LS included processes directly involved in fighting off the infecting pathogen, such as controlled cell death, controlling processes involved in normal muscle growth, correcting misfolded proteins, digesting dead cells, platelet activation and controlling energy metabolism. 

This contrasts with the immune and bleeding response in head-kidney, which were mostly focused on long distance cell signaling to control the immune response and inflammation, and production of new blood in response to prolonged bleeding from lesions. In summary, the ontology-based enrichment analysis of tDE-mRNAs indicated that the gDE-miRNAs likely regulated the protein expression of a large number of genes involved in different kinds of responses to the infection and the winter-ulcer lesions cause by *M. viscosa.*

We further explored the enrichment analysis results to reveal whether individual Reactome pathways were targeted by subgroups of gDE-miRNAs. The complete results from this search is given in Supplementary File S7. A summary of the main findings is shown in Table 10 and Table 11.

The general finding from this search was that most gDE-miRNAs were predicted to target at least one tDE-mRNA in almost all enriched gene pathways both in HK and LS materials. Although only eight of the gDE-miRNAs targeted genes in the two most enriched pathways (CO_2_/O_2_ exchange in erythrocytes and FOXO, Figure 3), they were not exclusive to these pathways. In summary, none of the gDE-miRNAs were associated with one or a few gene pathways only.

Despite being previously annotated as myomiRs [49], ssa-miR-1-3p, ssa-miR-133-1-4-5-3p, ssa-miR-133-2-3-3p and ssa-miR-206-3p were all gDE-miRNAs in head-kidney. Among these, ssa-miR-1-3p stood out as being one of only two gDE-miRNAs not predicted to play a role in stress response or cell cycle control, and ssa-miR-133-2-3-3p was the only gDE-miRNA not predicted to play a role in platelet activation. Despite having the same seed sequence as ssa-miR-206-3p, ssa-miR-1-3p targeted fewer tDE-mRNAs and was involved in fewer pathways. These two miRNAs shared 42 target genes, but while ssa-miR-1-3p only had one gene (beta-adducin) not targeted by ssa-miR-206-3p, ssa-miR-206-3p had 60 target genes not targeted by ssa-miR-1-3p (Supplementary Table S3).

## 3. Discussion

### 3.1. Methodological Considerations 

Three samples were used as controls in the LS materials. While they were from one time point only and the number is at the lower end of what is considered sufficient for estimates of biological variation in a comparative study of this kind, the results from the microarray analysis (DE-mRNAs and the following enrichment analysis) were in agreement with previous findings in similar challenge studies of *M. viscosa* and other bacterial pathogens [36] (discussed in Section 3.3), The fact that the protein coding genes identified as differentially expressed were indeed representative for infection with *M. viscosa*, lends support to number of samples (including controls) being sufficient for detecting gene expression changes due to *M. viscosa* challenge. It is therefore likely that the same holds true for the detection of differentially expressed miRNAs responding to *M. viscosa* challenge in the same materials.

Argonaut has a preference for mature miRNAs starting with U at the 5′ end [20]. Inspecting the mature sequences of the DE-miRNAs (Supplementary Table S1) the majority of those annotated as guide miRNAs did indeed have a U (T) as first nucleotide at the 5′ end (Supplementary Table S1). This fact supported that the threshold chosen in this study did discriminate correctly between guide and passenger mature miRNAs. In some cases (e.g., ssa-miR-132 or ssa-miR-2188), the differences in mature read counts between two mature miRNAs stemming from the same precursor were not large enough to define one as the major biologically relevant product, and both were included in the downstream target prediction analysis. However, although Argonaut has a strong preference for mature miRNAs with 5′ U vs. a 5′ C, Argonaut does incorporate mature miRNAs starting with other bases [20]. The few cases where the read counts from DE miRNAs originating from the same precursor were similar, and both were included in our downstream analyses, they were likely representing such cases where both 5p and 3p were biological relevant mature miRNAs. In summary, the 5′ base preferences of Argonaut for choosing gDE-miRNAs did support our annotation of guide miRNAs. As pointed out in Ramberg et al. [23], each mature miRNA has the potential to target hundreds of FL-mRNAs. The removal of the passenger miRNAs prior to downstream analysis, as carried out in this study, is therefore extremely important to avoid introducing errors in the final enrichment analysis of tDE-mRNAs. 

Enrichment analyses must be interpreted with caution. Here, we apply two kinds of enrichment analysis: homology-based enrichment analysis and genes identified as enriched in certain conditions applying the TM/TS framework. The latter does not identify gene function per se, but rather identify genes responding to a certain condition based on similar expression pattern in many similar studies (e.g., of bacterial infection). The limitation of this approach is that negative observations cannot be used to claim that certain genes are not involved in responses to certain bacteria or certain bacterial loads. However, a positive observation, e.g., if a gene is bacteria-responsive in a similar manner in many different experiments, including the enrichment analysis here, it lends credence to that gene being responsive to bacterial infection in general (not only a certain type of bacteria, time of sampling, etc.). The TM/TS enrichment approach may therefore supplement and support findings from homology-based enrichment analysis (which rely on the assumption that a certain gene has same function in Atlantic salmon as in a relatively distant model species). The TM enriched protocol also has the advantage that it can recognize genes responding to certain general conditions (e.g., bacterial infection) even if their functions are unknown. The effect of such genes being miRNA targets cannot be easily interpreted as their functions are not known. However, although their functions are unknown, they (either the genes or the miRNAs targeting these genes) may still represent valuable biomarkers for a given condition. Relatively few proteins have been experimentally studied to reveal their biological functions in Atlantic salmon. The TM enrichment protocol is also valuable in such a context as it points out proteins with unknown functions that clearly are associated with, e.g., bacterial infection, and as a consequence, would be very interesting to study further by appropriate experimental methods to disclose their functions.

### 3.2. Most of the gDE-miRNAs Are Conserved Vertebrate Mirnas Reported as Involved in Immune Responses, Inflammation, Angiogenesis and Wound Healing 

Our study identified multiple differentially expressed miRNAs. In the LS materials these gDE-miRNAs were associated with high bacterial loads and lesions that penetrated the basement membrane and displayed significant miRNA responses only at the very severe stage of the infection in muscle/skin tissues (Table 3). The HK materials revealed a slightly different pattern with a group of gDE-miRNAs showing an early response to infection, even before any bacteria could be detected in HK. Some of these showed a similar pattern at the late time point. Another group of HK gDE-miRNAs, showing either up or down regulation, were exclusively changed at the late time point (34 days post challenge, Table 4). Despite many gDE-miRNAs being identified in HK and LS, only miRNAs from three families (miR-132, -144 and -2188) were identified in both materials. This likely reflects that different miRNAs play different roles in response to bacterial infections in the head-kidney, an important immune organ in teleost, and at the primary site of infection at LS where the tissue damages occurred.

Among the gDE-miRNAs with an early response in HK, five (miR-15bf-5p [50], ssa-miR-23c-3p [50,51], ssa-miR-132-3p [13,50], ssa-miR-152-3p [50] and ssa-miR-724-5p [52]) have been identified as important in immune responses to bacterial infection in vertebrates. Additionally, miR-132 has been implicated in regulation of inflammation and wound healing [53], as well as antigen-dependent T-cell activation [54] and hematopoietic stem cell maintenance [55]. Notably, miR-132 was also reported as differentially expressed in viral challenge studies in Atlantic salmon [25,26,56]. Ssa-miR-15bf-5p was differentially expressed both during early and late stages of infection. This miRNA has been identified as playing a regulatory role in both inflammation as well as angiogenesis [57,58].

miR-1, -133, and -206 are all classified as myomiRs [49], meaning they are typically enriched in muscle tissues. Despite this, all three were differentially expressed (upregulated) both during early and late stages of infection in head-kidney, but not in the lesion site materials. While miR-1 and miR-206 have identical seed sequences [49], the additional mature miRNA sequence differences led miR-206 to have significantly more targets. Ssa-miR-1 had only one unique target gene (beta-adducin, which is involved in hematopoiesis) while ssa-miR-206 had 61 additional target genes. The myomiRs have all been found to play a role in tissue regeneration and miR-206 regulates inflammation in macrophages [13,49,59,60,61]. Interestingly, *M. marinum*, a pathogenic mycobacterium, induces upregulation of miR-206 in Zebrafish leading to reduced neutrophil response from the host. If *M. viscosa* acts in a similar manner, the repression of miR-206 could have a therapeutic effect on disease development [62].

Six gDE-miRNA families were responding only at the late stage of infection in the HK materials (Table 4). All of these have been shown as involved in regulation of immune responses, angiogenesis, or wound healing. The three downregulated miRNAs were; miR-137 (angiogenesis and muscle regeneration [63,64]), miR-218 (regulation of cytokines, chemokines and interferon production, hypoxia-responding [65,66,67]) and mir-489 (apoptosis and wound healing [68,69,70]). Similarly, the three upregulated miRNAs have been reported as involved in regulation of disease relevant responses; miR-730 is an Actinopterygii-specific miRNA responding to bacterial infection in Zebrafish [52,71,72]), miR-144 regulates apoptosis/cell cycle control [73] while the teleost specific miR-2188 responds to viral infection and is likely involved in macrophage differentiation [25,74]. 

The gDE-miRNAs identified in the LS materials were all responding to severe infection causing tissue damage. Again, a large number of vertebrate studies have reported the majority of these miRNAs as responding to viral and bacterial infections [13,24,25,26,50]. Some of the miRNAs well-described as pathogen-responsive from the LS materials included miR-23 (apoptosis, angiogenesis, muscle growth and regeneration [13,50,75,76]), miR-223 (regulation of inflammation in response to infection or muscle regeneration [13,50,77,78]), miR-125 and miR-146 (both annotated as viral as well as bacterial infection-responsive [13,32,50]) and miR-7132 (teleost-specific infection-responsive [25]). Two others of these gDE-miRNAs; miR-144 and miR-451 are expressed from a conserved miRNA gene cluster [17]. Both were reported as responding to bacterial infection in several teleost species and involved in apoptosis and cell cycle control regulation [13,24,73,79]. Another conserved gene cluster encoding miR-212 and miR-132 was also differentially expressed in the LS materials. These two miRNAs (miR-212 and miR-132) are involved in regulation of hematopoiesis [55].

Previous studies of miRNA responses to bacterial infection in Atlantic salmon are limited in number. However, one study of the bacterial pathogen *Piscirickettsia salmonis* did (although rather different criteria were used to define differential miRNAs) report miRNAs from the families ssa-miRNA-21, ssa-miRNA-146 and ssa-miRNA-451 as disease responding [28]. It is however noteworthy in this context that *Piscirickettsia salmonis* causes a less intensely ulcerative disease, with some fish not showing external signs of infection at the time of death [80], while as shown above many of the miRNAs identified in this study likely play a role in wound healing or response to blood loss, making it less surprising that these were not observed as having differentially expression in response to a different bacterial pathogen. The miRNA families miR-21 and miR-146 were also differentially expressed in response to both infection with viral pathogens [26], to infestation with sea lice and when Atlantic salmon was stimulated with pathogen-associated molecular patterns [29,32], suggesting they play very general roles in immune-response regulation. Similarly, miR-29, miR-155, miR-223, miR-2188 and miR-7132 have all been reported as differentially expressed in response to viral infections in Atlantic salmon [25,26,81], and thus likely have regulatory roles that are not specific to only bacterial infections. 

In summary, the comparisons to findings in other, similar studies of conserved vertebrate miRNAs’ involvement in response to bacterial infection as well as similar studies in Atlantic salmon supports that the association between gDE-miRNAs and *M. viscosa* infection reported here likely reflects their roles as mediators of immune responses, apoptosis, and regulators of angiogenesis and wound healing. 

### 3.3. Microarray Analysis Reproduced Findings from Prior M. viscosa Challenge Studies and Pointed out Uncharactherized Teleost Genes Important in Disease Response

There was a strong trend towards immune-responsive genes, both in terms of enrichment of functional categories and transcripts annotated as bacterial infection-responsive among the large number of DE-mRNAs identified in our study (Table 6 and Table 7). Some of the differentially expressed genes revealed the largest changes among the bacteria-responsive immune-genes. For example, in LS, catechol O-methyltransferase domain-containing protein 1, Macrophage inflammatory protein 2-alpha, Matrix metalloproteinase-9, complement C1q-like protein 2 and MMP 13 (Supplementary Table S2) all showed 150-200-fold higher expression in the high-infection category, and they are all reported as bacteria-responding immune genes [36]. In general, the findings here agreed very well with previous microarray studies on *M. viscosa* infection in salmon [35,36]. 

The STARS and GO/KEGG frameworks identified many genes as involved in immune response, wound healing, and other enriched disease response relevant gene pathways (Table 6 and Table 7, and Supplementary Table S2). However, annotation for many transcripts in non-model species like salmon is incomplete, especially for teleost specific genes where there are no well-annotated homologs in model species. The benefit of the TM/TS expression pattern-based annotation framework was that it suggested them as important responders to certain conditions despite their missing functional annotations. The uncharacterized transcripts LOC106568262 and LOC106585901, e.g., both show upregulated expression at Time Point 1 in HK which was increased at Time Point 2 (Supplementary Table S2). Results from the TM/TS annotation framework revealed these as upregulated genes important in response to bacterial infection (Supplementary Table S2). In all, there were 69 such uncharacterized genes (18 in HK, 45 in LS and 6 in both, Supplementary Table S2). These genes are of great interest, both because they likely have unknown immune or wound healing functions, and because they may reveal responses to bacterial infection that are unique to teleosts. 

A study of expression changes in *M. viscosa* lesion materials applying RNA sequencing was recently reported [37]. There are several differences between the current study and this RNA seq study (e.g., sampling time points, LS sites sampled, application of two completely different platforms (RNA seq and microarray)) which could lead to different results due to study design. The fact that the genes in the Atlantic salmon genome are often not well annotated and annotation of genes in the two studies was carried out by slightly different approaches also makes a detailed comparison difficult. However, if the same transcripts are detected in both studies this lends credence to them being important *M. viscosa*-responsive genes in LS tissues. On the other hand, if a transcript is not observed as differentially expressed in both studies they cannot be ruled out as false positives as there are so many differences in study design. Despite the mentioned differences in study design, we did carry out such a comparison, and the number of genes detected as differentially expressed in both studies was about one third of all genes detected in each of the studies. Limiting the enrichment analysis to only genes shared between both studies showed 59 pathways significantly enriched (Supplementary Table S8). These included many of the immune and lesion response pathways reported as enriched in the LS DE-mRNAs (e.g., cross-presentation of particulate exogenous antigens, detoxification of reactive oxygen species, platelet degranulation, neutrophil degranulation, interleukin-1 and -4 signaling, cytokine signaling in immune system, interferon gamma signaling, immune system, and hemostasis), adding further evidence that the shared genes play a role in *M. viscosa* infection response. 

In summary, the reproduced detection of *M. viscosa*-responding genes in this transcriptome study firmly establishes these genes, and those gene pathways where they were enriched, as important in response to winter-ulcer. The fact that the microarray analysis agreed very well with prior studies [35,36,37] also provide confidence that the investigation of miRNAs in same materials has the capacity to identify disease relevant miRNAs. 

### 3.4. Enrichment Analysis of tDE-mRNAs Supports That the gDE-miRNAs Are Key Regulators in Host Responses to M. viscosa Infection 

The in silico analysis applied to predict targets of the gDE-miRNAs (Supplementary Tables S3, S4 and S7) revealed many genes with known function associated with immune processes, inflammation, angiogenesis and wound healing. The finding that the majority of the conserved gDE-miRNAs were reported as involved in response to infectious diseases in different vertebrate species (including many viral pathogens in Atlantic salmon) (Section 3.2) further suggested that they, in a similar manner, were important in the host response to *M. viscosa*. The results from the enrichment analyses of tDE-mRNAs (Supplementary Tables S5 and S6) were also consistent with gDE-miRNAs being involved in regulation of gene pathways important in disease response and wound healing.

The TM/TS enrichment revealed that target genes were indeed overrepresented transcription modules and transcription signatures associated with response to bacterial infection. Comparing the enrichment analysis of all DE-mRNAs to the enrichment analysis of tDE-miRNAs showed that the target genes were not only enriched in the same TM and TS categories, but were in fact even more enriched in most of these categories (Table 5 vs. Table 9). The TM and TS categories matching tDE-mRNAs were those annotated as bacterial infection-responsive across multiple studies, as well as those belonging to TS categories relating to *M. viscosa* infection, infection by the ulcerative bacterial pathogen *Tenacibaculum*, wound healing, erythropoiesis and other immune and stress response signatures [36,39,40,45]. 

The Reactome Pathway overrepresentation analysis of tDE-mRNAs further supported that the gDE-miRNAs were regulating gene pathways involved in immune response, inflammation and other responses related to counteract the damage at the lesion sites (Figure 3 and Figure 4, and Supplementary Table S6). The differences in gene pathways enriched in the two materials likely reflected that miRNAs have different roles when responding to *M. viscosa* infection. The HK gDE-miRNAs primarily regulated processes related to immune signaling and hemostasis (Figure 3), while the LS gDE-miRNAs regulated processes involved in more direct immune responses and wound healing (Figure 4). While there was variation in the tDE-mRNAs predicted as targets of individual gDE-miRNAs (Supplementary Table S7), each gDE-miRNA targeted multiple tDE-mRNAs belonging to most of the target pathway categories. This supports earlier findings that each miRNA can have a multitude of targets, and each mRNA can be regulated by multiple miRNAs acting in concert [21,23].

In summary, this is the first study that aims to characterize miRNAs involved in -responses to *M. viscosa* infection. Multiple miRNAs were identified as differentially expressed in the two materials investigated. The majority of these miRNAs were recognized as involved in response to infection from similar studies in a number of vertebrate species. Furthermore, the in silico predictions of target genes and the following enrichment analysis of target genes suggested that these miRNAs were involved in regulation of a number of gene pathways important in host response to *M. viscosa* infection and in the healing process at the lesion sites. A large number of target mRNAs were predicted, some with unknown function, some targeted by teleost specific gDE-miRNAs, possibly representing novel and teleost-specific genes with important roles in host response to bacterial infection. The current characterization of gDE-miRNAs and their putative targets paves the way for future experimental validation studies. Such studies could expand our understanding of the mechanistic details and impact on host response to bacterial infection that are contributed by miRNA-guided post-transcriptional gene regulation in teleosts.

## 4. Materials and Methods

### 4.1. Moritella Challenge Trial and Tissue Sampling

The fish used in this study were from a challenge trial carried out at the controlled isolation facilities of VESO Vikan Research Station, Namsos, on Atlantic salmon (strain Stofnfiskur Optimal, from Benchmark Genetics Iceland HF (Hafnafjördur, Iceland) (Stofnfiskur HF prior to 2021)). The smoltified fish were acclimated to sea water for eight days prior to challenge. A total of 263 fish were included, 213 of which were challenged and 50 which were mock challenged controls. The fish were unvaccinated, and screened for antigens to other common viral and bacterial pathogens (e.g., *Aeromonas salmonicida*, *M. viscosa,* IPNV, and multiple varieties of *Vibrio*), prior to being used in the challenge trial. The average weight of the fish was 87.0 g at the initiation of the challenge trial. The fish were starved 1 day prior to challenge and on the day of challenge. Outside of this, the fish were fed to satiation before, during and after the challenge trial with the commercial fish feed Skretting Nutra Olympic 2.0 mm (Skretting, Stavanger, Norway). Feed was administered continuously (24 h per day).

**Figure 5:**
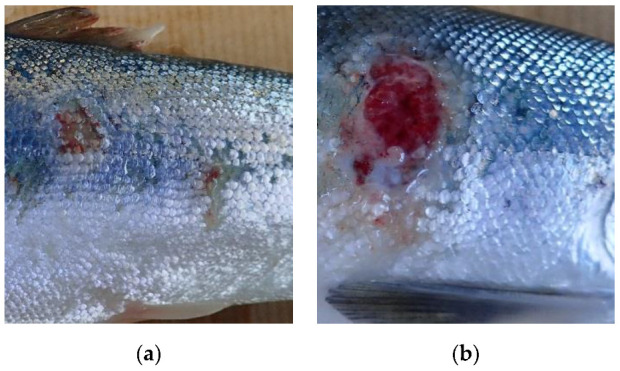
(**a**) Fish with multiple category 1 lesions. (**b**) Fish with one category 2 lesion.

Fish were bath challenged as described in Karlsen et al., 2017 [10]. Briefly summarized, infection was induced by the introduction of bacterial culture (*M. viscosa* strain VI-96/09/1016 VESO) to the challenge tanks to a final concentration 1 × 10^6^ cfu/mL. All tanks were closed for inlet and outlet of seawater flow, while oxygen saturation was maintained artificially. The challenge study was conducted over the course of 34 days, with temperatures ranging from 8–9.5 °C throughout the period. Mortality was recorded on a daily basis, and cause of death was confirmed by bacteriological sampling. Ten fish were collected from controls and challenge tanks at 9, 23, 34 days post-challenge (referred to as time points 1, 2, and 3, respectively). Additionally, 10 control fish were taken from the challenge tank prior to introduction of the bacterial culture. For each fish, samples were taken of the head-kidney (HK), as well as from the site of external ulcers, or from the abdomen if the fish did not exhibit visible lesions despite being infected (confirmed by *M. viscosa* qPCR, see Section 4.2). These samples contained both dermal and muscle tissue and are named lesion site (LS) samples for simplicity. Cutaneous lesions on each fish were described using the scoring system described in Karlsen et al. [10], with the following categories; 0: No visible lesions, 1: Superficial lesion that do not penetrate the basement membrane, or scale pocket oedema, 2: Lesion that penetrates the basement membrane, 3: The fish has died from the infection. Examples of categories 1 and 2 lesions are shown in Figure 5.

All fish handling procedures complied with the guidelines of the EU-legislation (2010/63/EU), as well as with Norwegian legislation. The bath challenge experiment and euthanization procedure were approved by the Norwegian Food Safety Authority under FOTS application nr. 11112. Dissection of fish and sampling of materials was in agreement with the provisions enforced by the Norwegian Animal Research Authority.

### 4.2. Total RNA Extraction and M. viscosa Analysis by qPCR

Total RNA was initially extracted from 140 samples (70 HK and 70 LS samples) using the *mir*Vana miRNA isolation kit (Ambion, Life Technologies, Carlsbad, CA, USA), in accordance with the manufacturer’s protocol. Extraction yield and RNA quality was measured on a NanoDrop1000 spectrophotometer (Nanodrop ND-1000, Thermo Fisher Scientific, Wilmington, DE, USA). RNA integrity (RIN value) was determined using an Agilent 2100 Bioanalyzer in combination with an Agilent 6000 Nano Chip (Agilent Technologies, Santa Clara, CA, USA). Following extraction, the total RNA was stored at −80 °C. Verification of infection was performed by PatoGen AS, Ålesund, Norway, according to their protocols. PatoGen is commonly used by Norwegian health authorities to measure M. viscosa load by qPCR. 

A number of samples did not meet our quality thresholds, and together with the Ct-scores from the qPCR measurements this formed the basis for the final selection of samples to include in miRNA and mRNA expression studies. A total of 46 samples were selected; 22 HK, and 24 LS, including controls, all with RIN values larger than 9.0 (Table 1 and Table 2). Going forward, the LS samples were grouped by CT score rather than time point, to maintain homogeneity within each of the groups being compared (see Results).

### 4.3. Library Preparation and Small-RNA Sequencing

Library preparation and sequencing of the samples was performed at the Norwegian High-Throughput Sequencing Centre (NSC; Oslo, Norway). The sequencing libraries were constructed using the NEBnext^®®^ multiplex small RNA Library Prep Set (New England Biolabs, Inc., Ipswich, MA, USA) according to the manufacturer’s protocol. The small RNA sequencing was carried out on an Illumina NextSeq 500 (Illumina, Inc., San Diego, CA, USA), which generated 75 bp single end reads. The sequencing data has been submitted to the NCBI Sequence Read Archive (SRA) under the Bioproject PRJNA830923.

### 4.4. DESeq2 Expression Analysis of miRNAs from Small-RNA Sequencing

Trimming of sequencing primers from the sequencing data was performed using the Cutadapt Python package (v.1.18 running on Python 2.7) [82], which also performed size filtering on the trimmed reads to only retain those between 18 and 25 nts in length. FASTQC (v.0.11.9) [83] was used before and after Cutadapt, in order to verify the effects of adapter trimming and size filtering, and to confirm that the final set of clean reads was of high quality (phred score 32 or more). The STAR aligner (v.2.5.2b) [84] was used to generate an index file from the currently known complete *Salmo salar* miRNAome [18], with default parameters apart from “--genomeSAindexNbases 6”, following the developer’s recommendation for indexing small datasets. The clean reads where then mapped unto the transcriptome index with STAR, applying default parameters apart from “--alignIntronMax 1”. The resulting BAM files were processed using the featureCounts function of the R package Rsubread (v.2.8.0) [85] with parameters countMultiMappingReads = TRUE and fraction = TRUE to generate a table of counts for each miRNA for each sample, fractioning multi-mapped reads evenly between each reported match for that read. 

The count tables were the input for differential expression analysis using the R-package DESeq2 (v.1.34.0) [86], which initially normalizes the samples in each group compared to allow for input datasets of varying sizes. For the head-kidney (HK) samples, the HK Control group was compared to samples from Time Point 1, Time Point 2, and Time Point 3 separately. The lesion site (LS) samples were divided in three groups differing in their infection severity based on results from the Moritella qPCR and lesion scores. The LS Control group was compared against CT > 30, CT 30–20, and CT < 20. miRNAs that passed a filter of baseMean >20, log2FoldChage either ≤ −1.0 or ≥ 1.0, and padj (Benjamini-Hochberg adjusted *p*-value) < 0.05, were categorized as Differentially Expressed miRNAs (DE-miRNAs). The mature DE-miRNAs were denoted as either guide DE-miRNAs (gDE-miRNAs) or passenger DE-miRNAs in the following manner: If one mature miRNA (5p and 3p) had 10 times or greater abundance (based on read count comparisons) than the other stemming from the same precursor, the one with much lower abundance was assumed to be the passenger miRNA [19,20], and therefore not included in the following in silico target predictions and enrichment analysis’. If there was less than 10 times difference in read counts between two DE-miRNAs processed from same precursor, they were both included in the downstream analysis (in silico target analysis and enrichment analysis). 

All DE-miRNAs as well as the subset defined as gDE-miRNAs were used for hierarchical clustering analysis with complete linkage and spearman correlation with an in-house R script employing the hclust function from the R package stats (v.4.1.3), along with the packages cluster (v.2.1.2), factoextra (v.1.0.7), and dendextend (v.1.15.2). Following clustering, heatmap.2 from the R package gplots (v.3.1.1) was used to generate heatmaps of the DE-miRNAs across compared groups, grouped by the hierarchical clustering analysis.

### 4.5. mRNA Microarray Analysis

The expression profiling to identify differentially expressed mRNAs (DE-mRNAs) in the HK and LS samples was performed at NOFIMA (Ås, Norway) with 44 k DNA oligonucleotide microarrays. The oligonucleotide microarray for Atlantic salmon was designed at Nofima. and based on 60-mer oligos for protein coding genes from the Atlantic salmon reference genome annotation (Salgeno-2, GEO accession #GPL28080) The bioinformatics pipeline STARS (Salmon and Trout Annotated Reference Sequences) [38] was used for annotation by functions (GO and custom vocabulary), pathways (KEGG) and expression changes under various conditions (TS and TM). Microarrays and reagents were manufactured by Agilent Technologies Inc., (Cedar Creek, TX, USA), and used in accordance with the manufacturer’s protocols. One-color hybridization was used, and each sample was analyzed with separate array. For HK, a subset of four samples were chosen to represent each group. For the LS samples, all three controls samples were compared against all four samples from the group LS CT < 20 (as this was the only group revealing DE-miRNAs). An overview of samples used for Microarray analysis are shown in Supplementary Table S2

Following hybridization and scanning, the microarray data was analyzed using the STARS pipeline [38], comparing each challenge group to the controls and identifying any mRNAs with log2 fold-changes ≤ −0.80 or ≥ 0.80 (1,75 fold change) and *p* < 0.05 (t-test) as Differentially Expressed mRNAs (DE-mRNAs). The STARS pipeline also automatically performed overrepresentation analysis of the transcripts in the KEGG, GO and STARs frameworks. The annotation of the Atlantic salmon genes detected by microarray oligos were based on their homology with model species, and same functions were assumed for the Atlantic salmon homologs. In addition to homology-based annotation, STARS also assigned transcription signature (TS) and transcription module (TM) annotations to each oligo and presented the most over-represented TS/TM categories. Genes were classified into a particular TS if they showed coregulation in a specific experiment in the Nofima Atlantic Salmon microarray database and were further subdivided into upregulated and downregulated TS. TM classifications were based on meta-analysis of the entire Nofima Atlantic Salmon microarray database, grouping together genes that show coregulation in response to one of four categories of experimental conditions: virus-responsive genes (VGS), bacteria-responsive genes (BACT), inflammation-responsive genes (INFL), and stress-responsive genes (STR) [36].

### 4.6. In Silico miRNA Target Prediction and Overrepresentation Analysis of Reactome Pathways in Predicted Target mRNAs

To facilitate use of the previously generated miRNA target prediction resource for Atlantic salmon, MicroSalmon (https://github.com/AndreassenLab/MicroSalmon/ (accessed 20 September 2022)) [23], the corresponding full-length mRNAs (Genbank Accession GIYK00000000) [22] of each DE-mRNA from the microarray analysis was identified in the following manner. The Oligo sequences of the DE-mRNAs were aligned to the FL transcriptome using the blastn tool in the BLAST+ package (v.2.9.0) [87], and FL sequences with at least one oligo sequence passing a filter of 90% sequence identity and 91% query coverage were classified as FL DE-mRNAs. The FL-DE-mRNAs and the DE-miRNAs from each of the two materials were used in conjunction with the MicroSalmon database to identify mRNA transcripts which were both differentially expressed and predicted to be targets of differentially expressed miRNAs, which were in turn were classified as FL-targets of a particular DE-miRNA (tDE-mRNA). The oligo IDs associated with each tDE-mRNA were processed in the STARS pipeline to identify overrepresented TM and TS categories in the subgroup of tDE-mRNAs.

The complete FL sequences, the predicted gene symbols and BLAST-derived natural language descriptions of the FL-targets from the transcriptome resource (Supplementary Tables S3 and S4) [22] were used as search input for the NCBI Gene database as well as the Universal Protein Resource UniProt (https://www.ncbi.nlm.nih.gov/gene (accessed 20 September 2022) and https://www.uniprot.org/ (accessed 20 September 2022), respectively), to identify gene symbols from human homologs. The non-redundant list of gene symbols of tDE-mRNAs were subsequently used as input in the PANTHER Overrepresentation Test (version 17.0 Released 12 February 2022) [88] (http://pantherdb.org/ (accessed 20 September 2022)) to identify gene pathways overrepresented in the tDE-mRNA dataset. The following parameters were used in the PANTHER Overrepresentation Test: *Homo Sapiens* REFLIST (containing 20,589 genes), and the Reactome version 65 Released 1 October 2021 annotation, and Fisher’s Extract and Calculate False Discovery Rate, retaining hits with an FDR < 0.05 and Fold Enrichment ≥2. The pathways were ordered hierarchically and the most specific pathway in each tree was retained. The exception to this was if the most specific pathway had the same number of representative genes as the next more general pathway above it in the hierarchy, in which case that one was retained instead. Finally, the specific pathways were grouped by manually assigned functional categories that were based on the pathways’ descriptions in the Reactome Pathway Browser (https://reactome.org (accessed 20 September 2022)) and the associated literature. The overrepresentation analysis results were plotted with ggplot using a publicly available R-script [89] to generate Figure 3 and Figure 4. 

## Figures and Tables

**Table 1:** Summary of HK samples, time points, RNA-extraction, *Moritella* measurement by qPCR, winter-ulcer-related lesion scores, and SRA accession numbers.

Sample ID	Category	DPC ^1^	A260/280	A260/230	RIN	Conc. (ng/µL)	qPCR Ct-Score ^2^	Lesion Score ^3^	Filtered Reads ^4^	NCBI SRA Accession
HK 1	HK Control	0	2.2	1.8	9.8	345	-	0	3,247,555	SRR18957529
HK 2	HK Control	0	2.3	1.9	9.8	419	-	0	5,321,144	SRR18957528
HK 3	HK Control	0	2.3	2.2	9.8	693	-	0	5,726,473	SRR18957517
HK 4	HK Control	9	2.2	2.2	9.7	233	-	0	5,504,990	SRR18957506
HK 5	HK Control	9	2.2	1.9	9.7	336	-	0	4,337,811	SRR18957495
HK 6	HK Control	9	2.3	2	9.6	860	-	0	4,807,673	SRR18957534
HK 7	T1	9	2.3	2	9.8	578	-	0	1,407,738	SRR18957533
HK 8	T1	9	1.7	1.4	9.7	725	-	1	4,844,452	SRR18957532
HK 9	T1	9	2.2	2.2	9.8	736	-	0	6,009,737	SRR18957531
HK 10	T1	9	1.6	1.5	9.6	881	-	1	3,626,493	SRR18957530
HK 11	T1	9	1.6	1.4	10	879	-	2	4,241,911	SRR18957527
HK 12	T2	23	2.3	1.5	10	680	29.2	2	3,786,326	SRR18957526
HK 13	T2	23	2.2	1.9	9.7	197	30	0	6,209,930	SRR18957525
HK 14	T2	23	2.1	2.3	9.7	257	35	1	6,338,451	SRR18957524
HK 15	T2	23	2.2	2.1	9.8	442	33	0	3,639,951	SRR18957523
HK 16	T2	23	2.3	1.8	9.8	742	35.4	0	3,712,448	SRR18957522
HK 17	T2	23	2.3	2.2	9.7	295	25.9	2	5,431,489	SRR18957521
HK 18	T2	23	2.2	2.3	10	525	32.1	2	4,058,169	SRR18957520
HK 19	T3	34	2.2	2.2	9.8	442	35.7	1	5,192,234	SRR18957519
HK 20	T3	34	2.2	1.7	9.7	445	35.3	1	3,209,097	SRR18957518
HK 21	T3	34	2.3	2.4	9.5	236	35.3	0	2,864,496	SRR18957516
HK 22	T3	34	2.3	2.2	9.1	878	36.2	0	8,072,161	SRR18957515

^1^ Days Post Challenge. ^2^ Cycle threshold scores for qPCR quantifying *M. viscosa* load in the samples. - = Not detected. ^3^ Categorization of sores caused by winter-ulcer disease as in Karlsen et al. [10] (see Methods). ^4^ Small RNA sequencing read count following adapter trimming and size filtering.

**Table 2:** Summary of LS samples, time points, RNA-extraction, *Moritella* measurement by qPCR, winter-ulcer-related lesion scores, and SRA accession numbers.

Sample ID	Category	DPC ^1^	A260/280	A260/230	RIN	Conc. (ng/µL)	qPCR Ct-Score ^2^	Lesion Score ^3^	Filtered Reads ^4^	NCBI SRA Accession
LS 1	LS Control	9	2.2	2.6	9.9	278	-	0	6,465,548	SRR18957514
LS 2	LS Control	9	2.2	1.8	10	275	-	0	6,022,915	SRR18957513
LS 3	LS Control	9	2.1	2.3	9.9	159	-	0	5,768,213	SRR18957512
LS 4	CT > 30	9	2.1	1.9	9.8	221	36.5	0	4,583,846	SRR18957497
LS 5	CT > 30	34	2.1	2.3	10	212	32.8	1	5,365,667	SRR18957496
LS 6	CT > 30	34	2.3	2	10	293	33.2	0	5,405,670	SRR18957494
LS 7	CT > 30	34	2.2	2.1	10	429	34.2	0	5,642,598	SRR18957539
LS 8	CT > 30	34	2.2	1.8	10	539	34	0	5,922,961	SRR18957538
LS 9	CT > 30	34	2.2	1.9	10	461	35.1	0	7,338,112	SRR18957537
LS 10	CT > 30	34	2.3	2.1	9.9	676	35.4	0	4,761,842	SRR18957536
LS 11	CT > 30	34	2.3	2.2	9.9	713	35.4	0	5,734,253	SRR18957535
LS 12	CT 20–30	9	2.2	1.9	9.9	303	26.3	0	5,893,412	SRR18957507
LS 13	CT 20–30	9	2.2	1.9	9.8	240	26.4	0	6,266,748	SRR18957505
LS 14	CT 20–30	9	2.2	2.1	10	461	24.5	1	6,131,531	SRR18957504
LS 15	CT 20–30	9	2.2	1.7	9.8	426	26.6	0	7,447,754	SRR18957503
LS 16	CT 20–30	9	2.2	2	10	245	25.6	1	7,537,766	SRR18957502
LS 17	CT 20–30	23	2.2	1.9	10	335	25.7	0	7,605,497	SRR18957501
LS 18	CT 20–30	23	2.2	2.1	10	264	29.2	0	5,431,896	SRR18957500
LS 19	CT 20–30	23	2.2	2	9.9	453	29.4	0	7,213,970	SRR18957499
LS 20	CT 20–30	23	2.2	2.2	9.9	415	29.2	0	6,017,065	SRR18957498
LS 21	CT < 20	9	2.2	1.8	9.6	311	17	2	6,539,457	SRR18957511
LS 22	CT < 20	23	2.2	2.2	9.9	403	17	2	6,750,776	SRR18957510
LS 23	CT < 20	23	2.2	2	9.3	131	15.5	2	5,780,356	SRR18957509
LS 24	CT < 20	23	2.2	2.2	9.5	395	16	2	5,118,308	SRR18957508

^1^ Days Post Challenge ^2^ Cycle threshold scores for qPCR detecting the presence of *M. viscosa* load in the samples. - = Not detected ^3^ Categorization of sores caused by winter-ulcer disease as in Karlsen et al. [10] (see Methods) ^4^ Small RNA sequencing read count following adapter trimming and size filtering.

**Table 3:** Overview of gDE-miRNAs in the CT < 20 group in LS.

Downregulated (Cluster 1)	Upregulated (Cluster 2)	Upregulated (Cluster 3)
ssa-miR-23b-3p ssa-miR-184-3p ssa-miR-193a-5p ssa-miR-205a-5p ssa-miR-205a-1-3p ssa-miR-205b-5p ssa-miR-499a-5p ssa-miR-499b-5p ssa-miR-551b-3p	ssa-miR-29a-5p ssa-miR-29a-3p ssa-miR-29b-2-5p ssa-miR-29c-5p ssa-miR-125b-1-3p ssa-miR-132-3p ssa-miR-144-5p ssa-miR-144-3p ssa-miR-146d-5p ssa-miR-212a-5p ssa-miR-451-5p ssa-miR-2188-5p ssa-miR-novel-2-5p	ssa-miR-21a-5p ssa-miR-29c-3p ssa-miR-122-5p ssa-miR-130d-1-5p ssa-miR-132-1-2-5p ssa-miR-138-1-2-3p ssa-miR-142a-5p ssa-miR-142b-3p ssa-miR-155-5p ssa-miR-223-5p ssa-miR-301b-5p ssa-miR-727b-3p ssa-miR-2188-3p ssa-miR-7132a-5p ssa-miR-7132b-5p ssa-miR-7132b-3p ssa-miR-novel-8-3p

**Table 4:** Overview of HK gDE-miRNAs and the time points where they showed significant expression changes.

Change	T1	T2	T3
Upregulated	ssa-miR-132-3p ssa-miR-152-3p ssa-miR-724-5p ssa-miR-23c-3p ssa-miR-1-3p ssa-miR-15bf-5p ssa-miR-133-1-4-5-3p ssa-miR-133-2-3-3p ssa-miR-206-3p	ssa-miR-23c-3p	ssa-miR-1-3p ssa-miR-15bf-5p ssa-miR-133-1-4-5-3p ssa-miR-133-2-3-3p ssa-miR-206-3p ssa-miR-144-5p ssa-miR-144-3p ssa-miR-730a-5p ssa-miR-2188-3p
Downregulated			ssa-miR-137bc-3p ssa-miR-218a-5p ssa-miR-218b-5p ssa-miR-489-3p

**Table 5:** Highly enriched TM and TS classifications among differentially expressed genes identified by microarray in Lesion Site and Head-Kidney materials.

Transcription Module/Signature ^1^	DE-Genes ^2^	TS/TM ^3^	Enriched	Reference ^4^
**Lesion Site Materials**				
TM Bacteria upregulated	399	524	7.65	[36]
TM Inflammation upregulated	112	170	6.62	[36]
*Tenacibaculum* dermis (up)	487	711	6.88	[36]
*Tenacibaculum* epidermis (up)	672	1260	5.36	[36]
*Piscirickettsia salmonis* head kindey (up)	947	2858	3.33	[36]
*Moritella viscosa* skin ulcer (up)	1410	4581	3.09	[36]
Wound healing skin (up)	651	1367	4.79	[39,40]
*Salmon alphavirus* heart (up)	342	919	3.74	[41]
Astaxinthin free diet muscle (up)	167	269	6.24	[42]
Plasmid injection muscle (up)	437	1284	3.42	[43]
**Head-Kidney materials**				
TM Bacteria upregulated	122	524	7.63	[36]
Vaccination days 14-35, head kidney (up)	109	169	21.12	[44]
*Moritella viscosa* spleen (up)	64	217	9.66	[36]
Critical swim test spleen (down)	70	241	9.51	[36]
*Tenacibaculum* epidermis (up)	90	372	7.92	[36]
Poly(I:C) head kidney(up)	39	141	9.06	[36]
Seawater adaptation gill (up)	77	358	7.04	Unpublished
*Tenacibaculum* dermis (up)	130	711	5.99	[36]
Respiration swim test heart (up)	71	343	6.78	[36]
Erythropoiesis spleen (up)	83	437	6.22	[45]

^1^ Names of enriched TM/TS categories. All TM categories have the prefix TM, all other categories are a transcription signature from a specific study given in the reference column, consisting of genes that were either upregulated or downregulated, as indicated in parathesis. ^2^ Number of differentially expressed genes identified on the microarray in this study assigned to the particular TM or TS. ^3^ Number of genes on the whole microarray assigned to the particular TM or TS. ^4^ References for the studies each individual TM or TS category is based on.

**Table 6:** Enriched STARS functional groups in LS.

Functional Category	Count ^1^	High Infection Fold ^2^
**Immune & defense responses**		
Immune receptors	18	**3.3**
Cytokine receptor	21	**4.5**
Antigen presentation	17	**2.1**
Acute phase	27	**2.5**
Complement	13	**4.4**
Lectin	27	**3.3**
TNF-related	48	**3.6**
Immune effectors	36	**3.0**
B cell	21	**3.1**
Lymphocyte	50	**4.4**
Immune proteases	10	**6.6**
T cells	18	**4.5**
Plasma proteins	20	**2.9**
Cellular stress	35	**2.4**
Protein folding & modification	43	**2.2**
Xenobiotic metabolism	35	**−1.8**
**Structures & processes**		
Lysosome	22	**1.6**
Proteasome	31	**2.3**
Keratin cytoskeleton	11	**−14.9**
Myofiber	51	**−1.8**
Peroxisome	7	**−3.8**
Mitochondria	324	**−2.2**
Retinoid metabolism	19	**−1.6**
Lipid metabolism	143	**−1.5**
Zinc metabolism	7	**2.4**
Differetiation homeobox	43	**−1.9**
ECM collagen	28	**−2.6**

^1^ Number of DE-mRNAs from the microarray annotated as belonging to the indicated functional category. ^2^ Mean log-2 fold change in expression compared to the controls of all the DE-mRNAs in the indicated category in the high infection group.

**Table 7:** Enriched STARS functional groups in HK. Significant differential expression is highlighted in bold.

Functional Category	Count ^1^	T1 Fold ^2^	T2 Fold ^2^	T3 Fold ^2^
**Immune & defense responses**				
Chemokines	14	−1.10	1.86	**−1.63**
Eicosanoid metabolism	8	**−1.42**	**−1.57**	1.07
Acute phase	9	−1.15	1.03	**−1.73**
Complement	6	−1.13	−1.08	**−1.63**
Ig receptors	6	**−1.47**	**−1.72**	−1.32
Lectins	12	−1.16	−1.14	**−1.53**
Lymphocyte	15	**−1.52**	**−1.53**	−1.12
T cell	14	**−1.40**	**−1.85**	−1.17
Ig	10	**−1.39**	**−1.58**	**−1.73**
Protein folding & modification	9	1.12	**1.43**	**1.43**
Redox homeostasis	10	**−1.35**	**−1.48**	**1.83**
Glutathione metabolism	5	−1.08	1.01	**2.04**
Xenobiotic metabolism	7	1.00	1.14	**1.41**
**Structures & processes**				
Cell cycle	31	1.20	1.21	**1.86**
Chromosome	20	1.12	1.05	**1.91**
Keratin cytoskeleton	6	**1.43**	**1.49**	**1.91**
Myofiber	36	**3.13**	**−1.55**	**1.83**
Mitochondria	33	**1.40**	**1.29**	**1.45**
Iron & heme metabolism	20	1.06	1.31	**2.03**
Globins	18	**−1.37**	**−1.39**	**3.53**

^1^ Number of DE-mRNAs belonging to the indicated functional category. ^2^ Mean log-2 fold change in expression compared to the controls of all the DE-mRNAs in the indicated category in each of the time point groups.

**Table 8:** Summary of data from identifying FL-mRNAs by use of microarray results (oligo sequences) and the following in silico target gene prediction.

Materials	Mapped Oligos ^1^	FL DE-mRNAs ^2^	tDE-mRNAs ^3^	Loci ^4^	Genes ^5^
HK	945	4223	2424	711	551
LS	3037	12.549	8965	2611	1917

^1^ Number of oligos identifying at least one FL-mRNA. ^2^ Total number of FL-mRNA isoforms identified by the oligo sequences ^3^ Number of predicted target genes among the FL-mRNA isoforms ^4^ Number of different loci transcribing the FL-mRNA isoforms ^5^ Number of different genes identified in the gene annotation process (several loci encoded paralogs).

**Table 9:** DEG counts and enrichment scores for TM and TS classifications that were highly enriched prior to target prediction in Lesion Site and Head-Kidney materials.

Transcription Module/Signature ^1^	tDE-Genes ^2^	TS/TM ^3^	Enriched	Reference ^4^
**Lesion Site materials**				
TM Bacteria upregulated	204	524	7.60	[36]
TM Inflammation upregulated	63	170	7.23	[36]
*Tenacibaculum* dermis (up)	261	711	7.12	[36]
*Tenacibaculum* epidermis (up)	387	1260	5.99	[36]
*Piscirickettsia salmonis* head kindey (up)	566	2858	3.86	[36]
*Moritella viscosa* skin ulcer (up)	796	4581	3.39	[36]
Wound healing skin (up)	367	1367	5.24	[39,40]
*Salmon alphavirus* heart (up)	187	919	3.97	[41]
Astaxinthin free diet muscle (up)	89	269	6.46	[42]
Plasmid injection muscle (up)	261	1284	3.97	[43]
**Head-Kidney materials**				
TM Bacteria upregulated	56	524	7.77	[36]
Vaccination days 14–35, head kidney (up)	53	169	22.80	[44]
*Moritella viscosa* spleen (up)	30	217	10.05	[36]
Critical swim test spleen (down)	33	241	9.95	[36]
*Tenacibaculum* epidermis (up)	49	372	9.58	[36]
Poly(I:C) head kidney(up)	26	141	13.40	[36]
Seawater adaptation gill (up)	38	358	7.72	Unpublished
*Tenacibaculum* dermis (up)	66	711	6.75	[36]
Respiration swim test heart (up)	36	343	7.63	[36]
Erythropoiesis spleen (up)	43	437	7.15	[45]

^1^ Names of enriched TM/TS categories. All TM categories have the prefix TM, all other categories are a transcription signature from a specific study given in the reference column, consisting of genes that were either upregulated or downregulated, as indicated in parathesis. ^2^ Number of differentially expressed target genes assigned to the particular TM or TS. ^3^ Number of genes on the whole microarray assigned to the particular TM or TS. ^4^ References for the studies each individual TM or TS category is based on.

**Table 10:** Distribution of the 17 HK gDE-miRNAs targeting at least one gene in the enriched functional categories and selected reactome pathways. Pathways indicated in italics below the category.

Functional Categories/Reactome Pathways	gDE-miRNAs
Immune system	17
*Interleukin signaling*	15
*Interferon signaling*	11
*SPM signaling*	10
Response to Stress	14
Platelet Activation	16
Hemostasis	17
*CO*_2_ */O*_2_ *exchange in erythrocytes*	8
Cell Cycle Control	14
*FOXO signaling*	8
Receptor Signaling	17

**Table 11:** Distribution of the 39 LS gDE-miRNAs targeting at least one gene in the enriched functional categories and selected reactome pathways. Pathways indicated in italics below the category.

Functional Categories/Reactome Pathways	gDE-miRNAs
Immune System	38
*Interleukin signaling*	38
*Interferon signaling*	35
*Cross-presentation of exogenous antigens*	17
Response to Stress	39
*Detoxification of reactive oxygen species*	27
Angiogenesis	39
Platelet Activation	36
Energy Metabolism	34
*Citric Acid Cycle*	28
Metabolism of Amino Acids	36
Cell Maintenance	39
*Mitochondrial biogenesis*	31
Cell Communication	39
Cell Cycle Control	39
*Programmed Cell Death*	37
Receptor Signaling	39
*GTPase activity*	39
Neuronal Growth	33

## Data Availability

All sequenced samples have been submitted to the NCBI Sequence Read Archieve Centre (SRA) (https://www.ncbi.nlm.nih.gov/sra (accessed 20 September 2022)) under Bioproject PRJNA830923. The microarray data has been deposited in NCBI’s Gene Expression Omnibus (https://www.ncbi.nlm.nih.gov/geo/ (accessed 20 September 2022)) and are accessible through GEO Series accession number GSE211004.

## Supplementary Materials

The supporting information can be downloaded at: https://www.mdpi.com/article/10.3390/ijms231911200/s1.
